# Importance of Tissue Doppler Evaluation in Dilated Cardiomyopathy: The Value of Diastolic Filling Pattern as a Prognostic Predictor

**DOI:** 10.3390/jcdd10060237

**Published:** 2023-05-28

**Authors:** Luminita Iliuță, Andreea Gabriella Andronesi, Marius Rac-Albu, Mădălina-Elena Rac-Albu, Alexandru Scafa-Udriște, Horațiu Moldovan, Florentina Ligia Furtunescu, Bogdan Constantin Rădulescu, Eugenia Panaitescu

**Affiliations:** 1 Medical Informatics and Biostatistics Department, University of Medicine and Pharmacy “Carol Davila”, 050474 Bucharest, Romania; 2 Cardioclass Clinic for Cardiovascular Disease, 031125 Bucharest, Romania; 3 Nephrology Department, University of Medicine and Pharmacy “Carol Davila”, 050474 Bucharest, Romania; 4 Nephrology Department, Fundeni Clinical Institute, 022328 Bucharest, Romania; 5 Department of Cardio-Thoracic Pathology, University of Medicine and Pharmacy “Carol Davila”, 050474 Bucharest, Romania; 6 Department of Cardiovascular Surgery, Clinical Emergency Hospital, 014461 Bucharest, Romania; 7 Academy of Romanian Scientists (AOSR), 050045 Bucharest, Romania; 8 Department of Cardiology, Clinical Emergency Hospital, 014461 Bucharest, Romania; 9 Department of Public Health and Management, Faculty of Medicine, ‘Carol Davila’ University of Medicine and Pharmacy, 050474 Bucharest, Romania; 10 C.C.Iliescu Emergency Institute for Cardiovascular Diseases, 022328 Bucharest, Romania

**Keywords:** dilated cardiomyopathy, diastolic dysfunction, restrictive pattern, prognostic predictors, remote monitoring, heart failure, COVID-19, telemedicine

## Abstract

(1) Background: The presence of restrictive left ventricular diastolic filling pattern (LVDFP) is associated with an unfavorable prognosis in many cardiac diseases, but few data are available on the prognostic implications of this pattern in patients with dilated cardiomyopathy (DCM). We aimed to establish the main prognostic predictors at the 1- and 5-year follow-ups in DCM patients and the value of restrictive LVDFP in increasing morbidity and mortality. (2) Methods: A prospective study of 143 patients with DCM divided in non-restrictive LVDFP group (95 patients) and restrictive group (47 patients). The patients were evaluated at a 5-year follow-up through an in-patient visit during the pre-pandemic period and hybrid methods (face-to-face, teleconsultation and home monitoring with a telemedicine application) during the pandemic period. Statistical analysis compared the two groups in terms of NYHA class, quality of life, hospitalizations/emergency department (ED) visits due to HF exacerbation and total mortality. (3) Results: The mortality rate in the restrictive group was markedly higher than that in the non-restrictive group at 1 year (17.02% vs. 10.59%, respectively, *p* < 0.05) and at 5 years (68.08% vs. 50.53%, *p* < 0.05). In the restrictive group, hospitalizations/ED visits due to HF decompensations at 1 year were significantly higher (85.11% vs. 57.89%, *p* < 0.05), with hospitalizations for ventricular arrhythmia being almost three times higher (21.28% vs. 7.37%, respectively, *p* < 0.05). The percentage of patients with a favorable evolution (in terms of NYHA class and quality of life) at the 1- and 5-year follow-ups were higher in the non-restrictive LVDFP group. The main prognostic predictors in patients with DCM at the 1-year follow-up were: restrictive LVDFP, age > 75 years, markedly dilated LV, comorbidities (DM, COPD), 2nd-degree mitral regurgitation and severe pulmonary hypertension (*p* < 0.05). (4) Conclusions: At the 1- and 5-year follow-ups, the presence of the restrictive LVDFP in DCM patients was independently associated with a poor prognosis, being the best clinical predictor for unfavorable evolution, after adjustment for other well-established predictive parameters in DCM patients.

## 1. Introduction

Dilated cardiomyopathy (DCM) represents one of the most important causes of morbidity and mortality among patients with congestive heart failure (HF). The evolution of this disease is often undulatory and, for this reason, difficult to predict both in the short term and in the medium and long terms. [1,2] Additionally, many of the patients have multiple comorbidities, requiring a multidisciplinary team for correct management [3]. Cardiologists are in a position to decide the best parameters to assess the severity of the disease and, consequently, improve the therapeutic management. Although great progress has been made in the recent years in terms of medical, interventional or surgical treatment, the general prognosis of this disease remains reserved, with an increased mortality at 5 years of approximately 50% [3,4,5,6], comparable to the mortality encountered in some of the most common neoplasias [2,3].

Previous studies revealed that the long- and medium-term prognoses in patients with DCM are influenced by many parameters, among which LV diastolic function is one of the most important [7]. This also appears to be the one of the earliest detectable abnormalities in many of the heart disorders.

Moreover, previous research has revealed that the restrictive LV diastolic filling pattern (LVDFP) is a strong predictor of poor clinical outcomes in most of cardiac diseases (valvular, coronary or congenital) [8,9,10,11,12]. There are few studies which evaluated the impact of restrictive LVDFP on the evolution and prognosis in patients with DCM. These studies are heterogeneous, with variable patient selection and a relatively small number, and most of them do not use the tissue doppler imaging (TDI) examination to assess diastolic function. However, there are no serial long-term studies on the evolution of LV dimensions and LV systolic and diastolic performance in DCM patients depending on the type of LVDFP.

To address this issue, we studied a series of patients with DCM using both clinical evaluation and echocardiography (including TDI), for 5 consecutive years.

The first objective of our study was to establish the main prognostic predictors at the 1- and 5-year follow-ups in DCM patients in terms of NYHA class, quality of life, hospitalizations/emergency department (ED) visits due to HF exacerbation and total mortality. Furthermore, we tried to assess the immediate and long-term prognostic implications of the type of LVDFP in patients with DCM and the value of restrictive LVDFP in increasing morbidity and mortality.

## 2. Materials and Methods

### 2.1. Study Population, Follow-Up Visits and Data Collection

The study population consisted of a prospective cohort of 142 patients with DCM, followed between 1 January 2018 and 1 January 2023 at a single center (Cardioclass clinic for cardiovascular disease, an ambulatory cardiology clinic, one of the few with a telemedicine application dedicated to follow up patients with HF). The patients enrolled in the study also participated in the development of the telemedicine application for HF during the COVID-19 pandemic [13]. Patients were eligible for enrolment into the study if they were diagnosed with DCM and had been evaluated in the clinic and registered in the dedicated application within the previous 12 months. A diagnosis of DCM was carried out in accordance with the definition provided by the Working Group on Myocardial and Pericardial Disease and ESC Guidelines, based on echocardigraphic examination [14]. The exclusion criteria were atrial fibrillation or other persistent cardiac rhythm alterations, ventricular-paced rhythm, left bundle branch block, need for mitral or aortic valve surgery, DCM secondary to other causes (pressure/volume overload, medication-induced, Chagas disease, Lyme disease), severe mitral regurgitation (MR), cardiogenic shock or LV assistance devices. Only 142 of the original 168 eligible patients with baseline DCM met all the criteria for inclusion in the present analysis.

All patients included in this study freely signed the informed consent form (that has been approved by the institutional ethics committee), in which they authorized the prospective collection of data for research purposes and the scientific analysis of their clinical data in an anonymous form.

The patients were evaluated clinically via echocardiography and laboratory parameters at the enrollment and during the follow-up, every 3 months before the pandemic, at least once yearly during the restricted period of the pandemic and at least twice yearly under the covering umbrella of vaccination.

During the pre-pandemic period (from 1 January 2018 to 29 February 2020), the standard follow-up of the patients consisted of in-person appointments (minimum of one appointment per trimester) with a cardiologist physician consultation, complete echocardiographic examination (including TDI) and/or ambulatory electrocardiogram (ECG) or blood pressure monitoring.

During the restricted pandemic period (from 1 March 2020 to 28 February 2021), the in-person appointments were drastically reduced (with no visits during the lockdown until 15 May 2020), these being limited to urgent situations and patients in NYHA classes III/IV. The follow-up consisted mainly of teleconsultations using a telemedicine application connected with our dedicated application, but we provided in-person visits with a full face-to-face evaluation at least once per year, including complete echocardiography. The home monitoring through the multiparametric platform (linked to our dedicated application) included the daily heart rate (HR), blood pressure (BP), body weight and symptom status. In addition, the application allows for weekly transmissions of electrocardiograms (ECG) (via the Istel HR-2000 remote monitoring system) and for the transmission of blood test results at least three times per year. The data collected through the telemedicine platform were evaluated and filtered by a specialized team of nurses and doctors, and based on them, medication changes were made, or patients who needed a face-to-face consultation were identified. Whenever it became necessary, patients with a poor response to oral diuretics and signs of congestion were admitted to our clinic for the administration of intravenous diuretics, based on a protocol established by the clinic doctors, that takes into account the current guidelines.

During the relaxed pandemic period (from 1 March 2021 to 1 January 2023), we provided a hybrid follow-up of the patients, with minimum two in-person appointments per year and teleconsultations (online or phone appointments and the tracking of the parameters entered in the telemedicine application).

Visits to the ED due to HF decompensation were defined as ambulatory day admissions to the observation room, with the need for intravenous diuretics (particularly furosemide), whereas hospitalizations were defined as admissions to the hospital for intravenous diuretics or to the intensive care unit for inotropic support.

Further, using teleconsultations and during the “face-to-face visits”, we evaluated the global quality of life using a customized self-reported questionnaire with two main aspects: the physical component (PCS) and the mental component (MCS). Patients self-assessed how their mental and physical quality of life evolved between two successive visits by answering the question: “How would you rate your quality of life now?”. The possible responses were “Better than previous visit”, “Same as previous visit” and “Worse than previous visit”. The points assigned ranged from 0 (lowest) to 10 (highest quality of life).

The most important endpoints used for the estimation of the short- and medium-term prognosis were: NYHA class of HF, hospitalizations or ED visits due to HF exacerbation, quality of life (appreciated on a scale from 1 to 10 using the self-reported questionnaire filled in by the patient at each visit) and death.

### 2.2. Ultrasound Methods, Variables of Interest

Echocardiographic examination was carried out at baseline at the enrollment into the study, during the pre-pandemic period every 3 months, and during the pandemic at least once yearly, using a Philips Affinity30 or a portable General Electric VIVID machine, with a 3.5 MHz probe for all examinations. All techniques, measurements and calculations were in accordance with the recommendations of the European and American Society of Echocardiography [15].

For each patient, we assessed the dimensions of the heart cavities (left ventricle (LV) end-systolic and end-diastolic diameters and volume, left atrium (LA) diameters, including the LA indexed volume) and LV systolic and diastolic performance (including complete TDI evaluation) [15].

A standard two-dimensional (2D) echocardiographic study was performed for the assessment of diastolic and systolic LV diameters (from the parasternal long axis view) and LV wall thickness. The LV end-diastolic volume and end-systolic volume were measured from apical four- and two-chamber views, applying the Simpson method, with is the calculation of the left ventricular ejection fraction (LVEF).

For the evaluation of diastolic function, we record the transmitral inflow profile with the pulsed wave Doppler (PW) placed between the mitral leaflet in an apical four-chamber view. The transmitral flow velocities (peak early diastolic velocity (E wave) and late diastolic velocity (A wave)) and the deceleration time (DT) were measured. In the same apical four-chamber view, we placed the PW sample volume in the lateral mitral annulus for the main TDI measurements (peak annular systolic velocity (Sa), early diastolic velocity (Ea) and late diastolic velocity (Aa)). 

The diagnosis of DCM was performed using ESC guideline criteria for DCM diagnosis [15]: LV dilatation (>112% corrected to body surface area and age) with a reduced function (shortening fraction < 25% and/or LVEF < 45%). Severely dilated LV was defined as the LV end-systolic volume > 95 cm^3^ or end-systolic diameter > 55 mm. For the DCM diagnosis, we did not take into account the NT-pro-BNP levels. 

The restrictive LVDFP was defined based on pulsed wave Doppler examination as the E wave deceleration time (DT) less than 150 msec or the E wave–A wave velocity ratio (E/A ratio) ≥ 2 or isovolumetric relaxation time (IVRT) < 60 ms, and based on TDI examination as Ea/Aa < 1 and E/Ea > 12.

For the diagnosis of the ischemic etiology of DCM, coronary angiography or coronary angio-CT was carried out in all patients over 35 years of age and in patients under 35 years old with angina pectoris. Sixty-nine patients had associated coronary artery disease (50% reduction in luminal diameter of any coronary artery) without an indication for revascularization. We measured the BNP for all patients at least 3 times per year and considered the cut-off of 300 pg/mL for HF diagnosis [16].

We calculated the mean pulmonary artery pressure using the tricuspid regurgitation velocity–time integral method. We defined pulmonary hypertension (PHT) as the mean pulmonary artery pressure more than 30 mmHg, and severe PHT as the mean pulmonary artery pressure more than 50 mmHg.

### 2.3. Statistical Analysis

Statistical analysis used Statistical Package for the Social Sciences, version 23.0, for the regression analysis and calculation of the correlation coefficient and relative risk.

Categorical variables are expressed as absolute numbers (percentages) and continuous variables with a normal distribution are presented as the mean  ±  standard deviation. Non-normal distributions are presented as the median (interquartile range). 

Qualitative data were tested using the Pearson Chi-square test, likelihood ratio and Fisher’s exact test and quantitative data between the two groups, with an independent sample *t*-test. 

Univariate logistic regression analysis was used to identify the potential independent predictors of unfavorable evolution (death or hospitalizations/ED visits due to HF decompensations) in DCM patients. After the univariate estimations were calculated, the odds ratios (OR) were obtained in multivariate models, including significant independent variables. Stepwise multivariate logistic regression analysis was performed to identify clinical and ecographical predictors for an unfavorable evolution. The area under the receiver operating characteristic (ROC) curve and the Hosmer–Lemeshow goodness-of-fit statistic test were calculated to assess the discrimination and calibration of the model, respectively. To evaluate the goodness-of-fit of the model, the Cox and Snell/Nagelkerke value was calculated. A *p*-value of <0.05 was considered statistically significant. 

For further estimations of the risk, we used Cox proportional hazards modeling by introducing all univariate predictors in a stepwise procedure into a survival multivariate model, with the entry and remove set to a significance level of 0.05. The variables with a low prevalence or exhibiting multicollinearity did not enter into the multivariate model. The proportional hazards model and interaction assumptions were tested and no violation was observed. A significant improvement in model prediction was based on the likelihood ratio statistic, which follows a chi-square distribution, and the *p*-value was based on the incremental value compared with the previous model. We identified few differences among the CIs of ORs obtained from the logistic regression and CIs estimated by Cox regression; that is why we reported mainly the OR values.

The main parameters tested as the risk factors for mortality and hospitalizations/ED visits due to HF decompensations in regression models were:

- age > 75 years;
- male gender;
- the presence of a restrictive LVDFP;
- LVES diameter > 55 mm;
- LVES volume > 95 cm^3^;
- LVEF < 25%;
- the presence of comorbidities (DM, COPD);
- the presence of 2nd-degree mitral regurgitation;
- the presence of pulmonary hypertension (PHT);
- ischemic etiology of DCM;
- paroxysmal atrial fibrillation;
- NYHA class IV;
- NT-proBNP > 10,000 pg/m.

## 3. Results

### Demographic and Clinical Characteristics of the Patients

A total of 164 patients with DCM were eligible for the study; in the end, the studied group included 142 patients (after applying the exclusion criteria or due to the fact that they were absent from the follow-up visits). A flowchart with the patients included and excluded in the study is presented in Supplementary Figure S1. The demographic, clinical, echographic characteristics of the patients and the treatment for HF are presented in Table 1.

Depending on the LVDFP, patients were divided into two groups:

- Group A—95 patients with non-restrictive LVDFP
- Group B—47 patients with restrictive LVDFP

All patients were treated with the standard medication for HF, including beta-blockers, digitalis, diuretics, angiotensin converting enzyme inhibitors and spironolactone. At the time of enrolment, all the patients were in sinus rhythm.

The main significant predictors for the presence of restrictive LVDFP in DCM patients revealed by univariate logistic regression analysis were:

- age > 75 years (OR = 1.75, 95% CI [1.28, 2.78]);
- diabetes mellitus (OR = 4.85, 95% CI [2.79, 8.56]);
- COPD (OR = 4.57, 95% CI [1.45, 12.78]);
- arterial hypertension (OR = 2.03, 95% CI [1.98, 3.92]);
- paroxysmal atrial fibrillation (OR = 3.79, 95% CI [1.76, 7.98]);
- ischemic etiology of DCM (OR= 2. 23, 95% CI [2.78, 4.08]);
- LVEF < 25% (OR = 3.78, 95% CI [2.45, 3.99]);
- NYHA class > III (OR = 4.88, 95% CI [2.58, 8.97]);
- severe PHT (OR = 2.92, 95% CI [1.45, 4.97]).

In the restrictive LVDFP group, the percentage of the patients with comorbidities (arterial hypertension, DM, COPD) was higher compared with the non-restrictive group.

The mortality rate at the 1- and 2-year follow-ups was significantly higher in the restrictive LVDFP group (17.02% vs. 10.59% in the non-restrictive group for the first year of follow-up, *p* < 0.05, respectively, 23.40% in restrictive group vs. 9.47% in the non-restrictive group for the second year of follow-up, *p* < 0.05), regardless of the LV systolic performance.

Moreover, hospitalizations and ED visits due to heart failure (HF) decompensations were higher in the restrictive LVDFP group (85.11% vs. 57.89% in the non-restrictive LVDFP group in the first year of follow-up (*p* < 0.05)). Hospitalizations for ventricular arrhythmia were three times higher in the restrictive group (21.28% vs. 7.37% in nonrestrictive group, *p* < 0.05).

Regarding the patients’ clinical course, the percentages of those with a favorable evolution, quantified as an NYHA class of HF less than 3 and self-reported quality of life score more than five at the one year follow-up, were higher in the non-restrictive LVDFP group. At the 1 year follow-up, the percentage of patients with a better or the same quality of life score was significantly higher in the non-restrictive LVDFP subgroup of patients compared with the restrictive one (58.13% vs. 13.04%, *p* < 0.005, likelihood ratio). At the 1-year follow-up, the percentage of patients in an NYHA class less than 3 was fourfold higher for patients with non-restrictive LVDFP (42.1% in non-restrictive LVDFP patients vs. 10.52% in restrictive LVDFP group, *p* < 0.05).

In the medium–long term, at the 5-year follow-up, the mortality rate was significantly higher in patients with restrictive LVDFP (68.08% in Group B) compared to patients with non-restrictive LVDFP (50.53% in Group A), regardless of the LV systolic performance or dimensions.

At the 1 year follow-up, the univariate predictors of unfavorable outcomes (death and hospitalizations/ ED visits due to heart failure (HF) decompensations) in the study cohort revealed by logistic regression and COX regression analysis (with similar odds ratios (OR), respectively, hazard ratios (HR)) were:

- the presence of a restrictive LVDFP (OR = 6.75, 95% CI [4.64–8.78]);
- age > 75 years (OR = 5.8, 95% CI [3.88–8.12]);
- LVES diameter > 55 mm (OR = 4.52, 95% CI [3.59–6.59]);
- LVES volume > 95 cm^3^ (OR = 5.32, 95% CI [3.64–7.78]);
- the presence of comorbidities (DM, COPD) (OR = 6.52, 95%CI [3.87–8.33]);
- the presence of 2nd-degree mitral regurgitation (OR = 5.41, 95% CI [3.74–7.89]);
- the presence of pulmonary hypertension (PHT) (OR = 2.8, 95% CI [1.58–3.89]).

When evaluated in a univariate model, restrictive LVDFP was associated with a worse outcome. The presence of the restrictive LVDFP has significantly increased the risk of death at the 1 year follow-up, regardless of the presence of other parameters known to increase mortality in DCM patients. 

Similar analysis was also performed after stratifying the patients on the basis of their LVEF, and the survival rate remained dependent on the type of LVDFP in DCM patients. Multivariate logistic regression analysis revealed that the restrictive LVDFP pattern turned out to be the main independent predictor for increasing the risk of death or hospitalization for HF decompensations (*p* = 0.001), regardless of the LV dimensions or performance, the presence of a secondary hemodynamically significant MR or severe PHT. Furthermore, the prognosis of the patients with the restrictive pattern was the worst, no matter the other factors involved. 

**Figure 1:**
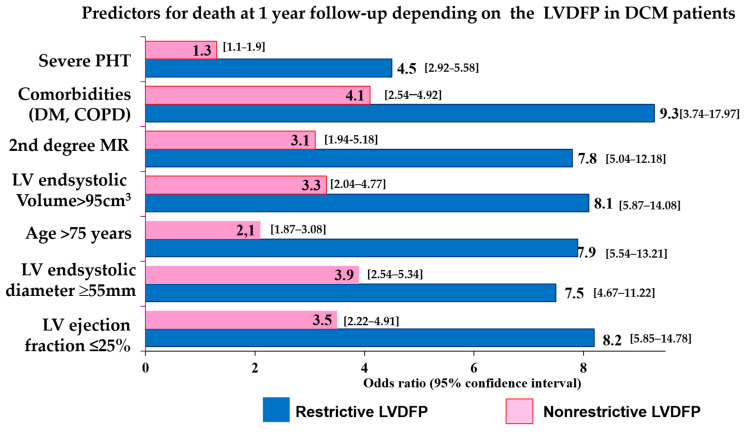
The early risk of death depending on the type of LVDFP in patients with DCM. LVEDSD—left ventricle end-systolic diameter; LVEF—left ventricle ejection fraction; LVDFP—left ventricle diastolic filling pattern; DCM—dilated cardiomyopathy, DM—diabetes mellitus; COPD—chronic obstructive pulmonary disease.

In Figure 1, the relative risks are presented distinctly depending on the type of LVDFP. The predictive value for death at the 1-year follow-up of the LV systolic performance or dimension, the patient’s age or of the presence of comorbidities, second-degree MR or severe PHT, was higher in patients with non-restrictive LVDFP. In these patients, the values of the LV ejection fraction less than 25%, markedly dilated LV with LV an end-systolic diameter > 55 mm and LV end-systolic volume > 95 cm^3^, age > 75 years, the presence of comorbidities (DM, COPD), and the presence of second-degree MR or severe PHT increased the risk of death at the 1-year follow-up (OR = 4.5–9.3, 95% confidence interval 1.1–17.97), the odds ratios values being higher in restrictive LVDFP group compared with the non-restrictive group.

**Figure 2:**
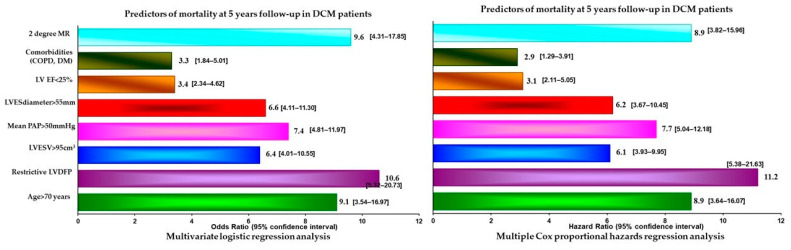
Predictors of mortality at the 5-year follow-up in patients with DCM. MR—mitral regurgitation; LVESV—left ventricle end-systolic volume; LVEDD—left ventricle end-diastolic diameter; LVEDV—left ventricle end-diastolic volume; LVESV—left ventricle end-systolic volume; PAP—pulmonary arterial pressure; DM—diabetes mellitus; COPD—chronic obstructive pulmonary disease.

At the 5-year follow-up, the main parameters associated with an unfavorable evolution in DCM patients revealed by stepwise multivariate logistic regression analysis and Cox proportional hazards analysis were: patient age more than 75 years, significantly dilated LV (end-systolic volume > 95 cm^3^, end-systolic diameter > 55 mm), restrictive LVDFP, 2nd-degree MR, comorbidities, LVEF < 25% and severe PHT (*p* < 0.0001) (Figure 2).

To adjust for differences between the groups and in order to determine whether LVDFP is an independent predictor for adverse outcome, we performed multiple Cox proportional hazards analyses. In a multivariate analysis in which all univariate (*p* < 0.05) predictors of outcome were considered, restrictive LVDFP remained a strong predictor of prognosis in DCM patients (hazard ratio HR = 4.48; 95% CI [2.28–6.78]; *p* = 0.008), independent of clinical data and standard Doppler echocardiographic predictors of outcome.

The other independent predictors of outcome at the 5-year follow-up were patients aged more than 75 years, significantly dilated LV (end-systolic volume > 95 cm^3^, end-systolic diameter > 55 mm), restrictive LVDFP, 2nd-degree MR, comorbidities, LVEF < 25% and severe PHT (*p* < 0.0001) Although LVEF was not a significant univariate predictor of the outcome, we included it in the multivariable model due to its clinical importance, but it was not a predictor of prognosis.

## 4. Discussion

Data from the present study support the hypothesis tested in other previous studies which highlighted the importance of LV diastolic filling as a predictor of severity and prognosis in DCM [17,18]. Restrictive LVDFP is found in patients with DCM, especially in those with severe forms of the disease, and is one of the best predictors of mortality and unfavorable evolution in terms of repeated hospitalizations for HF [19,20,21,22]. Thus, in our study, in patients with DCM with restrictive LVDFP, both at the 1-year and 5-year follow-ups, the mortality rate was significantly higher compared to patients with a non-restrictive filling pattern. Additionally, in terms of the NYHA class and quality of life, at the 2- and 5-year of follow-ups, they were clearly superior to the group with non-restrictive LVDFP, the same results being obtained in other studies [17,18,23,24,25,26,27].

Although previous reports showed that gender may have a very strong impact on echocardiographic and clinical outcomes in DCM patients, we did not find significant differences between the sexes. Other studies showed that women affected by DCM experienced better outcomes compared to men. Alongside that, females experiencing left ventricular reverse remodeling showed the best outcomes in the long-term follow-up.

A study which evaluated the prognostic implications of the evolution of restrictive LVDFP in DCM patients concluded that the persistence of restrictive filling at 3 months is associated with a high mortality and transplantation rate. On the other hand, patients with reversible restrictive filling had a higher probability of improvement and excellent survival [19,20,21,22].

There are few studies taking into account the type of LVDFP in DCM patients. Pinamonti et al. showed that restrictive LVDFP is frequent in DCM and is associated with more severe disease, being a powerful indicator of the increased mortality risk and need for heart transplantation [28,29].

Fantini et al. examined the main factors possibly involved in the resolution or persistence of restrictive LVDFP after surgical ventricular restoration in a series of patients with ischemic cardiomyopathy, and found that restrictive filling was reversed after surgical ventricular reconstruction in almost 50% of the patients studied, and was associated with an improved NYHA class [30].

In all published studies, the survival rate at 2 years was higher in patients with a non-restrictive filling pattern compared to patients with restrictive LVDFP [19,20,21,22,26,27,28,29,30]. The survival rate was 84%, 73% and 61% at 1, 2 and 4 years, respectively, in another study dealing with DCM patients, that is significantly lower compared to that of age- and gender-matched population. [29]. At the 2-year follow-up, we found a mortality rate in the study group with standard treatment, slightly higher than those from the literature, probably because of the underuse of novel treatments, such as sacubitril, and because the transplantation program is not well developed in our country.

The risk of death at the 1- and 2-year follow-ups was increased by older age, the important enlargement of the LV, the presence of comorbidities, severe MR and severe PHT, as shown in previous studies [7,10,20,21,22,25,26,27,28,29,31,32,33,34,35,36,37]. 

We observed that in spite of the survival of patients with restrictive LVDFP, their quality of life was much worse compared to those with the non-restrictive pattern. Severe systolic dysfunction of the LV has a lesser influence upon evolution compared to restrictive LVDFP. Thus, echocardiography, and especially TDI, for the evaluation of LV diastolic performance turned out to be one of the best investigations in order to stratify patients with DCM in terms of HF hospitalization, NYHA class, quality of life and mortality.

More advanced imaging techniques, such as cardiac magnetic resonance imaging, might provide more precise details regarding LV structure and dimensions in DCM patients. Furthermore, restrictive LVDFP was the main baseline parameter associated with a poor prognosis in this series of patients in a logistic model, that included age, LVEF, LV dimensions, MR degree, comorbidities and PHT [38]. 

We demonstrated that the echocardiographic evaluation of the LV diastolic performance and the diagnosis of restrictive LVDFP is reflected in the clinical status at the follow-up visits, when patients with non-restrictive filling showed a significantly better NYHA functional class and a trend toward fewer adverse clinical events compared with the restrictive LVDFP patients. A larger series of patients must be investigated to establish whether the restrictive LVDFP represents the main medium- and long-term prognostic predictor in patients with DCM.

### Study Limitations 

There are some limitations of our study: it is a single-center observational study, with a small sample size, a medium follow-up period and a low number of events. 

Doppler-derived LV filling pattern can be influenced by multiple factors, such as HR, loading conditions, paced rhythm and left-sided valvular disease. We excluded patients with chronic atrial fibrillation, moderate-to-severe mitral or aortic valve disease and those with a pacemaker. Furthermore, we did not evaluate Valsalva maneuver-related changes in the PW Doppler findings, which are useful in differentiating pericardial constriction and myocardial restriction.

The lack of cardiac magnetic resonance imaging data made it impossible to investigate the eventual relationship between LVDFP changes during the follow-up period and the extent of baseline ischemia and replacement fibrosis [38,39]. Moreover, we did mot have information on right-heart catheterization, although previous studies reported the diagnostic and prognostic utility of right-sided catheterization and endomyocardial biopsy in idiopathic dilated cardiomyopathy [40].

On the other hand, we had a reduced number of ICDs, which is explained by the fact that access is still insufficient in our country and those who have these devices are generally followed in the hospitals where they were implanted and not in the outpatient setting. Since access to heart transplant or LV assisting devices is very limited in our country, we did not include these parameters among the study outcomes.

A lot of patients are on digitalis because it is very accessible in our country, other more expensive treatments (such as sacubitril/valsartan) being more difficult to access.

## 5. Conclusions

In patients with DCM, the presence of a restrictive LVDFP is associated with a more unfavorable prognosis. This type of filling increased the risk of death and hospitalizations for HF decompensations, and worsened the clinical status of the patients (quantified as NYHA class and quality of life). In addition, for prognosis evaluation in DCM patients, a more reliable parameter is LV diastolic performance, independent of clinical data and standard Doppler echocardiographic predictors of outcome.

At short- and medium–long terms, the presence of a restrictive LVDFP, second-degree MR, dilated LV with LVESD > 55 mm and LVESV > 95 cm^3^, and severe PHT can imply higher mortality rates in DCM patients.

## Figures and Tables

**Table 1:** Baseline clinical, ecographic and demographic characteristics of the patients *N* = 142.

	Group A—95 pts Non-Restrictive LVDFP	Group B—47 pts Restrictive LVDFP	*p*-Value
Mean (SD) age (years)	59 (11)	63 (13)	0.381 ^1^
Women, no. (%)	41 (43.1%)	19 (40.4%)	0.584 ^2^
Medical history, no. (%)			
Arterial hypertension	49 (51.6%)	27 (57.4%)	0.229 ^2^
Diabetes mellitus	39(41.1%)	36 (76.6%)	0.001 ^2^
Paroxysmal atrial fibrillation	32 (33.7%)	26 (55.3%)	0.035 ^2^
Ischemic etiology of DCM	45 (47.4%)	24 (51.1%)	0.059 ^2^
Chronic kidney disease	32 (33.7)	15 (31.9%)	0.338 ^2^
COPD	11 (11.6%)	14 (29.8%)	0.012 ^2^
Mean (SD) LVEF (%)	32 (5)	22 (4)	0.003 ^1^
LVEF ≤ 25%, no. (%)	19 (20%)	18 (38.3%)	0.001 ^2^
Mean (SD) heart rate (b/min)	75 (17)	84 (18)	0.641 ^1^
Mean (SD) systolic blood pressure (mm Hg)	125 ± 18	105 ± 12	0.052 ^1^
NYHA^a^ class I/II, no. (%)	40 (42.1%)	5 (10.6%)	0.001 ^3^
NYHA^a^ class III, no. (%)	39 (41.1%)	25 (53.2%)	
NYHA^a^ class IV, no. (%)	13 (13.7%)	20 (42.5%)	
Median NT-proBNP (IQR) ^b^ (pg/mL)	1192 (800–2693)	1929 (800–2693)	0.034 ^1^
Medications, no. (%)			
ACEi or ARB	60 (63.2%)	29 (61.7%)	0.114 ^3^
Sacubitril/valsartan	35 (36.8%)	11 (23.4%)	0.212 ^3^
Beta-blocker	88 (92.6%)	40 (85.1%)	0.071 ^3^
Mineralocorticoid receptor antagonist	90 (94.7%)	44 (93.6%)	0.511 ^3^
Ivabradine	10 (10.5%)	3 (6.38%)	0.442 ^3^
Digitalis	55 (57.9%)	26(55.32%)	0.125 ^3^
Diuretic	53 (55.8%)	37(78.73%)	0.041 ^3^
Implantable cardioverter defibrillator	2 (2.1%)	3 (6.38%)	0.001 ^3^

LVEF—left ventricle ejection fraction; LV—left ventricle; NYHA—New York Heart Association; ACEi—angiotensin-converting enzyme inhibitor; ARB—angiotensin receptor blocker; COPD—chronic obstructive pulmonary disease. ^a^ New York Heart Association (NYHA) class reflects the patient status during the last face-to-face pre-pandemic appointment. Plus–minus values are the means ± standard deviation. 1. Independent sample *t*-test; 2. Pearson chi-square; 3. Likelihood ratio. ^b^ NT-pro-BNP denotes N-terminal pro-B-type natriuretic peptide plasma levels, expressed as pg/mL, and IQR represents the interquartile range.

## Data Availability

All data generated or analyzed during this study are included in this published article.

## Supplementary Materials

The following supporting information can be downloaded at: https://www.mdpi.com/article/10.3390/jcdd10060237/s1, Figure S1: Flowchart regarding inclusion-exclusion criteria.

## Abbreviations

LVDFP—left ventricle diastolic filling pattern; DCM—dilated cardiomyopathy, LV—left ventricle; LVEF- left ventricle ejection fraction; DT—deceleration time, NYHA—New York Heart Association; ED—emergency department; DM—diabetes mellitus; COPD—chronic obstructive pulmonary disease; MR—mitral regurgitation; COVID 19—Coronavirus disease 2019; HR—heart rate; BP—blood pressure; HF—heart failure; ACEi—angiotensin-converting enzyme inhibitor; ARB—angiotensin receptor blocker; LVES—left ventricle end-systolic; LVEDD—left ventricle end-diastolic diameter; LVEDV—left ventricle end-diastolic volume; PAP—pulmonary arterial pressure; EDt—E-wave deceleration time; IVRT—isovolumetric relaxation time; TDI—tissue doppler imaging.
