# Supplementary figures and images for: Importance of Tissue Doppler Evaluation in Dilated Cardiomyopathy: The Value of Diastolic Filling Pattern as a Prognostic Predictor
Source: J Cardiovasc Dev Dis. 2023 May 28;10(6):237. doi: 10.3390/jcdd10060237 (PMC10298846; doi:10.3390/jcdd10060237)

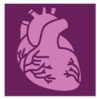

Figure 1 Supplemental. Flowchart regarding inclusion-exclusion criteria

1

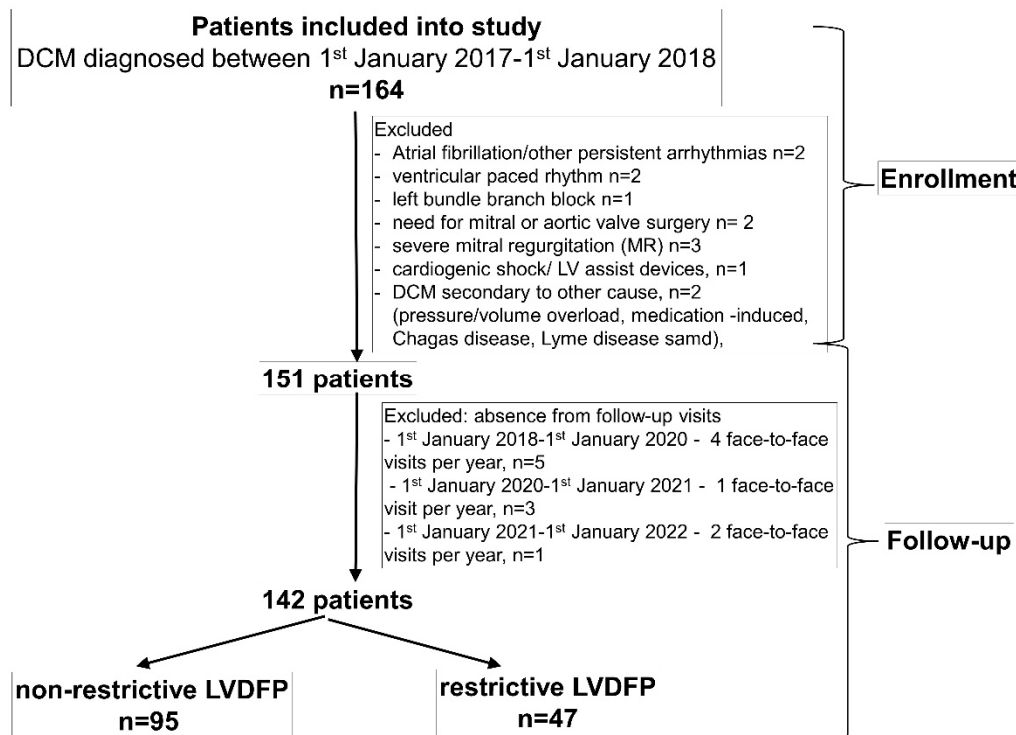

2

3

4

Supplement: Supplementary file 1 [file jcdd-10-00237-s001.zip › jcdd-2369642-supplementary.pdf]
